# Investigating the Driving Factors of Public Participation in Public-Private Partnership (PPP) Projects—A Case Study of China

**DOI:** 10.3390/ijerph19095192

**Published:** 2022-04-25

**Authors:** Ziqian Luo, Junjie Li, Zezhou Wu, Shenghan Li, Guoqiang Bi

**Affiliations:** 1 School of Business, Macau University of Science and Technology, Macau 999078, China; 2109853xbs20002@student.must.edu.mo; 2 Underground Polis Academy, Shenzhen University, Shenzhen 518060, China; lijunjie2018@email.szu.edu.cn (J.L.); shenghan@szu.edu.cn (S.L.); 3 Key Laboratory for Resilient Infrastructures of Coastal Cities (Shenzhen University), Ministry of Education, Shenzhen University, Shenzhen 518060, China; 4 Sino-Australia Joint Research Centre in BIM and Smart Construction, Shenzhen University, Shenzhen 518060, China; 5 Jinan Haiying Real Estate Development Company, Jinan 250000, China; guoqiang5663@163.com

**Keywords:** Public-Private Partnership, public participation, driving factor, China

## Abstract

Public participation is an important procedure of the environmental impact assessment. Effective public participation is essential to the Public–Private Partnership (PPP) projects as such projects usually exert tremendous impacts on the environment and society. However, in literature, there are few studies investigating the driving factors of public participation in PPP projects, especially in the context of China. To bridge this research gap, this study proposed a theoretical model, which incorporates contextual factors (i.e., perceived benefit and perceived risk) into the classical Theory of Planned Behavior model, to explore the determinants. The initial proposed model was tested using structural equation modeling. Analysis results indicated that attitude towards behavior, subjective norm, perceived risk and perceived behavioral control were the four significant driving factors of public participation in PPP projects, whereas perceived benefit had limited impact. Furthermore, this study evaluated eight public participation approaches in PPP projects. Results revealed that the public were more willing to participate in public decisions through the internet platform, followed by the information disclosure or consultation provided by the government. The research findings derived in this study can provide valuable reference for the government to promulgate proper policies to attract more public participation in PPP projects. Moreover, the research idea and methods used in this study can be popularized in other countries to enhance the public participation in PPP projects.

## 1. Introduction

With the increase of social demand for infrastructure projects and public services, local governments are facing huge pressure of lacking sufficient governmental budget [1,2]. As a potential solution of this problem, Public–Private Partnership (PPP), which is a form of partnership contract, has been proposed and adopted by governments around the world [3]. In general, PPP can be described as a cooperation between public sectors and private sectors to meet public needs through appropriate risk allocation and benefit sharing [4,5,6]. From this point of view, it is worth stating that PPP is a mode of cooperation based on the public interests. Thus, it is important to maintain a balanced relationship between public sectors and private sectors, and the fulcrum of this balance is the end user of the project, the public [7,8]. In addition, PPP projects usually have far-reaching social and environmental impacts; thus, public participation should be fundamental and essential to avoid potential adverse effects in PPP projects [9].

In the environmental impact assessment procedures, public participation is an integral part [10,11,12,13,14]. Nevertheless, a PPP project involve many stakeholders who may have various concerns from different aspects. In the actual implementation, as PPP projects are not equal in benefits across varied social groups and the public cannot participate in the contracting process, the rights and interests of the public could be easily ignored to some extent [15,16,17]. The lack of public participation in PPP projects is widespread in many developing countries and a series of social contradictions and conflicts have been arisen [18,19,20]. For example, in China, a waste incineration power plant was announced to be established in Panyu district; however, this decision was questioned and protested by the local residents for its inadequate environmental impact assessment. To solve this problem, the local government further engaged in various programs which involve the local residents to deal with the conflicts, and the social crisis was finally solved [21,22]. The occurrence of these incidents not only aroused public doubts towards the PPP mode, but also made negative impact on the credibility of the government. In order to better implement the PPP mode, Osei-Kyei and Chan [23] claimed that mobilizing public participation in PPP projects is an effective solution.

In recent years, China has been developing PPP projects at a fast pace. By the end of January 2019, the number of PPP projects in China was 8788, involving 19 industries such as transportation, ecological environment, education, medical care, and district development [24]. However, as reported by Zhou, et al. [25], the public participation mechanism of PPP projects in China is not satisfactory and the degree of public participation is quite low. The enthusiasm and effectiveness of public participation are affected and restricted by many factors [26,27]. Thus, identifying the driving factors are the premise to improve public participation in PPP projects.

The aim of this paper is investigating the driving factors of public participation in PPP projects in the context of China. The remainder of this paper is organized as follows. The Section 2 provides a literature review on the public participation in PPP projects and the Theory of Planned Behavior. The Section 3 describes the proposed theoretical model. Then, the questionnaire design and data collection process are presented. Structural Equation Modeling is used to test the proposed hypotheses. The analysis results are presented and discussed in the Section 5 and Section 6. Finally, this paper is ended with a conclusion.

## 2. Literature Review

### 2.1. Existing Research on Public Participation in PPP Projects

Public participation is a basic right of citizens, which embodies the basic principle of fairness and reasonableness in a democratic society. Nowadays, public participation has been applied in various activities, such as waste disposal [28], urban planning [29] and environmental impact assessment (EIA) [30,31]. Studies in these activities can provide significant references for public participation in PPP projects. For instance, the psychological and physiological characteristics of the public (such as age, gender, etc.) may affect their participation behavior [32]. Meanwhile, the lack of relevant professional knowledge and ability would have an impact on the willingness to participate [33]. When participation channels and opportunities are insufficient, public participation will also be affected [34].

Nowadays, as PPP has been extensively employed in practice, scholars gradually realized the importance of public participation in PPP projects [35]. Studies have been made to investigate effects of public participation on the success of PPP projects. For example, Demirag [36] emphasized the participation of end users and found that increasing public participation had an important impact on the economic benefits of the projects. Afterwards, Majamaa, et al. [37] constructed a Public–Private–People Partnership (4P) model based on the perspective of rational consumption and publicity from the perspective of end-user, urging public sectors and private sectors to better understand public needs and provide more valuable products or services. Ng, et al. [38] further established a bottom-up public participation mechanism based on the 4P model by using the stakeholder theory, which could better realize the goal of infrastructure construction and operation.

In China, the public participation in PPP projects have also been emphasized in recent years; however, relevant research was mainly conducted from an economic perspective. For example, Li, et al. [39] explored the influence of the public participation of the PPP projects supervision behavior, and suggested improving the public participation level and influence through the application of the Internet. Song, et al. [40] evaluated the effective thresholds of public participation in adjusting the cooperative behaviors of both the government and the private investor. Wang and Gao [41] further revealed that the increase of public satisfaction could assist in improving the overall effectiveness of PPP projects. Recent research conducted by Li, et al. [42] confirmed that the public’s active participation could achieve a win-win situation of economic and environmental performance.

As can be concluded from previous research, public participation could be beneficial to achieve the effectiveness of PPP projects; however, from the literature review, it was found that the current research in the field of public participation mechanism is not enough. Few studies have examined public participation from the perspective of the formation of behavioral intention. To fill this research gap, this study chose behavioral intention as a focus to investigate the driving factors and influencing paths of public participation in PPP projects, and attempted to put forward the guiding measures to promote public participation in PPP projects. The research findings are expected to contribute in promoting the motivations of public participation in PPP projects.

### 2.2. Theory of Planned Behavior

**Figure 1:**
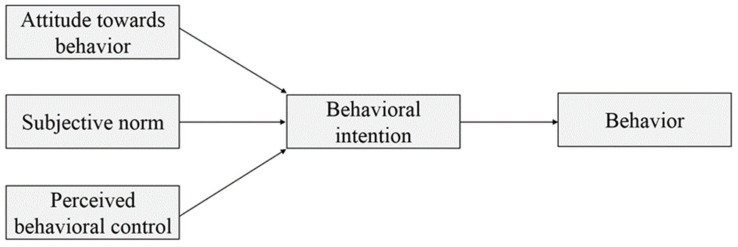
Theoretical model of Theory of Planned Behavior (Ajzen, 1991).

The Theory of Planned Behavior (TPB) was originated from the Theory of Reasoned Action (TRA), which is one of the most important theories in social psychology to explain and predict personal specific behavior. It details the most immediate factors that influence an individual behavior. According to TPB, a personal behavior is positively influenced by his/her behavioral intentions, and these intentions are influenced by three factors: (1) attitude toward behavior (i.e., individual positive or negative assessment of a behavior); (2) subjective norm (i.e., individual perception of the expectations of important people or groups); and (3) perceived behavioral control (i.e., individual perception of the conditions required for his/her successful behavior) [43]. The framework of the TPB is shown in Figure 1.

The TPB has been widely applied since it was put forward. At present, it has been applied in many fields, such as waste management behavior, low-carbon tourism behavior and so on [44,45,46]. A large number of studies have proved that the TPB in individual behavioral research has strong predictive accuracy. However, while being affirmed and supported, the TPB has also been questioned. For example, Bagozzi and Nataraajan [47] argued that the TPB only regards behavior as a goal to discuss, and the theoretical factors have ignored the role of goal in the decision-making process. Some scholars questioned whether the factors of the TPB are sufficient to fully explain the individual behavior and intention, and tried to add some new factors to the theoretical model in order to improve the explanatory power [48,49,50]. These challenges and doubts promote the development and perfection of the TPB. Therefore, when applying the theoretical model in practice, some modifications could be made on the basis of the fundamental model to better explain the practical research problems. 

In existing literature, the TPB has been applied in the field of public participation and proved to be effective to explain relevant behavior intentions. For example, Turcanu, et al. [51] studied public participation intention related to nuclear research facilities. Martin, et al. [52] investigated the drivers of public participation in marine citizen science. Ma, et al. [53] identified the determinants affecting the public intention to participate in waste recycling programs. However, in the field of public participation in PPP projects, the application of TPB for investigating the determinants remains unexplored. Thus, this study explored the driving factors of public intention to participate in PPP projects on the basis of the classical TPB.

## 3. Theoretical Model Development

**Figure 2:**
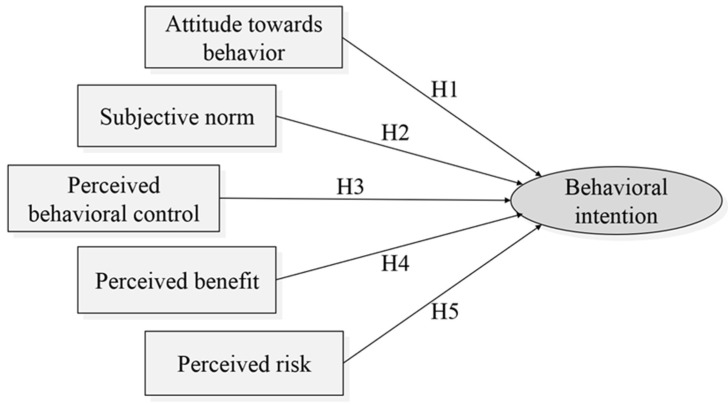
The preliminary theoretical model.

Figure 2 shows the TPB-based theoretical model based on a comprehensive literature review. In addition to the general framework of the TPB, other new factors are also introduced in the proposed model. The hypothesized relationships among the factors in the model are discussed below.

### 3.1. Attitude towards Behavior

Attitude towards behavior refers to a personal positive or negative evaluation of an action before taking it (Ajzen, 1991). A person who believes that behavior can bring positive results tends to show a positive attitude towards behavior. In the TPB, attitude towards behavior can directly affect individual behavioral intention, which is able to predict individual behavior in a certain extent [54]. Many scholars believe that attitude towards behavior is the most important factor affecting individual behavioral intention, and this effect is reflected in many cases [55,56]. If the public hold a more positive attitude towards participating in PPP projects, then their intention of participating in these projects may be stronger [57]. In other words, only the public think that participating in PPP projects is necessary and effective, and the more they would participate in these projects. Therefore, the following assumption is put forward.

**Hypothesis** **1** **(H1).** *Attitude toward behavior has a direct positive effect on the behavioral intention of the public to participate in PPP projects*.

### 3.2. Subjective Norm

Subjective norm refers to the degree to which important individuals (family members, friends, etc.) and organizations (governments, community organizations, etc.) put forward opinions and suggestions that affect the personal decision to carry out an action (Ajzen, 1991). It reflects the degree of individual perception of external pressure [58,59]. Harrison [60] pointed out that the subjective norm affecting behavior in the TPB may originate from individuals who worship, respect or believe that their suggestions are credible. In public participation activities, when the public decides whether to participate in PPP projects decision making or supervision, perceived social pressure may affect the formation of their intention to participate. It reflects the pressure or influence of important individuals or groups on public participation in the project process (Ajzen, 1991). Generally speaking, the greater the perceived external support, the more likely the public is to participate in the behavior, so as to adapt themselves to the expectations of the surrounding individuals or groups. Therefore, the following assumption is proposed.

**Hypothesis** **2** **(H2).** *Subjective norm has a direct positive effect on the behavioral intention of the public to participate in PPP projects*.

### 3.3. Perceived Behavior Control

From the framework of the TPB, it can be seen that perceived behavior control is an important factor affecting behavioral intention, which aims to measure the behavior of individuals who are not completely controlled by will (Ajzen, 1991). It reflects an individual past experience and anticipated obstacles to a particular behavior, and is the individual perception of the difficulty level in performing a certain behavior [61]. According to the TPB, the more resources and opportunities individuals believe they possess, the higher their perceived behavioral control, and consequently their intention to act [62,63]. However, public participation in PPP projects is not a simple process of participation, but a behavior that would be constrained by many objective factors or their own conditions [64]. When public individuals perceive that they have less obstacles to implementation and expectations in the process of decision making or supervision in PPP projects, their intention to participate in the behavior may be stronger. Thus, the following hypothesis is proposed.

**Hypothesis** **3** **(H3).** *Perceived behavioral control has a direct positive effect on the behavioral intention of the public to participate in PPP projects*.

### 3.4. Perceived Benefit

Perceived benefit refers to the benefit that people feel a product can provide for them [65]. Past studies have shown that perceived benefit had a direct positive correlation with consumer behavior [66,67,68]. Hsu and Lin [69] believed that perceived benefit directly or indirectly affected consumers’ attitudes, and then influenced purchase intention, which was an important factor affecting transactions, and can drive purchase behavior. Since the public is the direct consumer of public goods or public services provided by PPP projects, the public intention of choosing whether to participate in PPP projects is similar to that of consumers’ choice of whether to buy products. The public can perceive the benefits of the projects and evaluate the value of participating in these projects. When the perceived benefit of the public is more in line with their own expectations and actual situation, the behavioral intention of participating in PPP projects may be stronger. Thus, from the perspective of consumers, this external factor is properly introduced into the model, and the following hypothesis is proposed.

**Hypothesis** **4** **(H4).** *Perceived benefit has a direct positive effect on the behavioral intention of the public to participate in PPP projects*.

### 3.5. Perceived Risk

In the field of public behavior, many scholars have studied perceived risk extensively [70,71,72]. The research showed that perceived risk was a major explanatory factor of public behavior. Similarly, Mitchell [73] believed that perceived risk was one of the most important concepts in understanding the public choices. In this study, perceived risk represents the public judgment on the risk in PPP projects. With the improvement of public awareness of rights protection, in the face of projects that may pose a threat to their own interests, they will generally participate in project decision making or supervision out of safeguarding their own rights and interests, attempting to eliminate risks or eager to obtain certain compensation. For the public, the living environment, health, fees and so on are the project risk factors that the public attaches great importance to, because they are closely related to the daily life. In some studies of not-in-my-backyard (NIMBY) facilities, scholars have pointed out that perceived risk would affect the behavior of public participation [74,75]. In addition, due to the long cycle of PPP projects, there may be a risk cost unfavorable to the public from construction to operation. When the public can perceive the project risks are larger, the behavioral intention of participating in PPP projects may be stronger. Thus, this external factor is also introduced in the model, and the following hypothesis is proposed.

**Hypothesis** **5** **(H5).** *Perceived risk has a direct positive effect on the behavioral intention of the public to participate in PPP projects*.

## 4. Methodology

The research methodology could be mainly summarized as four stages, such as initial questionnaire design, questionnaire refinement, data collection and data analysis. The detailed procedures of the stages are presented as follows.

### 4.1. Survey Instrument

A questionnaire was designed to investigate the driving factors of public participation in PPP projects. The questionnaire consisted of three parts: (1) background information of the respondents, including gender, age, educational background and type of work unit; (2) measurement items of extended TPB model; (3) an open question inviting the respondents to put forward more suggestions and ideas for this study. The questionnaire was pretested by 20 persons. The feedback was positive, so no major changes were made. Some minor changes were made due to suggestions on wording. As an example, “I think public participation can reduce public opposition” was revised to “I think public participation can improve the public understanding of PPP projects and help the projects go smoothly”.

### 4.2. Measurement Items

The respondents were required to use the 5-point Likert scale ranging from 1 (strongly disagree) to 5 (strongly agree) to rate the measurement items. The 5-point Likert scale was utilized because its usage rate is higher than other scales and it could produce more reliable evaluation results [76,77]. The measurement items in the official questionnaire were listed in Appendix A and described as follows.

According to Ajzen [78], attitude towards behavior was measured by asking respondents to evaluate the consequences of participating in PPP projects. The measurement items mainly include previous research on the benefits of public participation in PPP projects, such as reducing the government’s decision-making mistakes, improving the public understanding of PPP projects, monitoring the behavior of the government and enterprises and so on [79]. 

The measure of subjective norm was operationalized referring to Zhang, et al. [80] by asking respondents to rate the extent to which “significant individuals or organizations” (including family, neighbors, residents’ community, government and media) would approve of their participation in PPP projects. 

Evaluation of the perceived behavioral control involved five items derived from Miśkowiec and Gorczyca [81] and Fares, et al. [82]. Perceived behavioral control was composed of personal situation and external conditions [83]. The personal situation was measured by the item about time, energy and cognitive ability [84]. The external conditions were measured with the item “I have sufficient ways/I can obtain relevant information to participate in PPP projects” [85]. 

The construct of perceived benefit involved five items derived from previous studies. These items include the potential impact of PPP projects on local life. For example, the better the local public infrastructure and services are, the higher the housing price is likely to be [86]. Besides, PPP projects in tourism, education, transportation and other fields will bring positive changes to the local basic necessities of life, which are closely related to the public [87,88,89]. 

The construct of perceived risk was based on five items. These items often occur in our daily life, which are potential risks for public dissatisfaction and opposition. For example, NIMBY PPP projects may affect the physical or mental health of the public [90]; unreasonable charges may exist in PPP transportation projects [91]; and some PPP projects are built without considering local customs probably, so as to destroy the culture [92].

Four items were selected to measure the behavioral intention referring to Richardson, et al. [93] and Nadlifatin, et al. [94]. These four items include decision making, supervision, participating in the early stages and recommending people around us to pay attention to PPP projects.

### 4.3. Data Collection

The theoretical model and the hypotheses were tested by SEM. In SEM, there is a certain requirement for the sample size that should not be too small, because such analysis is sensitive to it [95]. Mueller [96] suggested that at least 100 cases should be used in the implementation of SEM, and greater than 200 cases would be better.

In this study, the survey population of the questionnaires were the public. Questionnaires were published online through Sina Weibo, WeChat and relevant public forum websites. However, online survey is easy to omit some hard-to-reach public groups to fill out the questionnaire [97], so the survey also used the convenience and snowball sampling. As the name implies, sample elements are identified by convenience (friends) and recommendation networks. When it is difficult to get response from randomly selected sample elements, this sampling method is preferred [98,99,100]. In this survey, persons such as government officials that are difficult to contact in the online survey are investigated through this method. This sampling method enables us to obtain more questionnaires economically and quickly from different public groups. Through the whole data collection process, a total of 282 responses were collected. Then the filtering process was carried out to ensure the quality of the response. The invalid questionnaires were filtered out according to two principles: (1) the completion time of the questionnaire was less than 5 min; (2) the answers were chosen with significant regularity in the questionnaire. After the process of filtering, 221 replies were remained, representing 78.3% of the total responses. 

The statistical information of the respondents is shown in Table 1. Men and women made up half of the respondents, respectively. The majority of respondents were aged 20 to 29, accounting for 76%. This distribution is reasonable as we think young people are more likely to accept online questionnaires; meanwhile, as the main force of social development in the future, they may have higher enthusiasm for public participation. Besides this, nearly 52.5% had a bachelor’s degree and 38.5% had a master’s degree or above. A total of 6.8% of the respondents worked in government departments, 12.2% in public institutions and 35.7% in state-owned or private enterprises.

### 4.4. Data Analysis

The collected data were analyzed by employing SEM via the Amos 22.0 software. SEM is a statistical method based on covariance matrix to analyze the relationship between variables. Moreover, it is an extension of general linear model, normally including measurement model and structural model [101]. For the sake of evaluating the model fitting, we adopted the two-stage model construction process recommended by Singh, et al. [102]. The first step is to evaluate the measurement model, and then to evaluate the structural model. 

The reliability and validity of latent factors were assessed firstly when evaluating the measurement model. Reliability is related to the internal consistency of the structure, and Cronbach’s α coefficient is utilized to measure the internal consistency of the measurement items [103]. Therefore, after exploratory factor analysis, the reliability of each latent factor can be tested by calculating the Cronbach’s α coefficients via the SPSS software [104]. Generally, the coefficient higher than 0.7 indicates a high reliability [105]. After that, confirmatory factor analysis was performed to verify the validity of the observed variables, which validity refers to whether the observed variables actually measure the structure that the researchers intend to measure. If the observed variables are significantly loaded on the hypothetical latent factors, or at least moderately loaded, they will be valid [106]. Var [107] suggested the observed variables will be valid if the factor loading coefficients are more than 0.5. 

After completing the above steps, the next step is to measure the structural model and improve its goodness-of-fit. The structural model was improved by using the modified indicators and removing the unimportant paths. Once the optimization model is obtained, the driving factors and regression weights can be ascertained and all hypotheses can be tested by the statistical significance of the estimated normalized path coefficients.

## 5. Results

This section introduces the results of SEM based on the collected data, including the measurement model and the structural model. The hypotheses are tested on this basis.

### 5.1. Measurement Model

Reliability evaluation was implemented in the first place. The coefficient α for each construction are reported in Table 2. As can be seen from it, the coefficients of all latent factors had exceeded the recommended level of 0.7.

Then, the validity of latent factors was verified by confirmatory factor analysis (CFA). The observed variable would be deleted while its factor loading was less than 0.5. Through the CFA, the observed variable of PBC5 was deleted because its factor loading was 0.43. After deleting PBC5, the CFA of all constructs were accepted. As shown in Table 2, all loading coefficients met the statistical significance at a confidence level of 0.001, and the loading factors of the items were more than the recommended level of 0.5 as proposed by Var (1998). Therefore, as shown in Table 3, it can be concluded that the measurement model is in good agreement with the data according to the goodness-of-fit indices.

### 5.2. Structural Model

**Figure 3:**
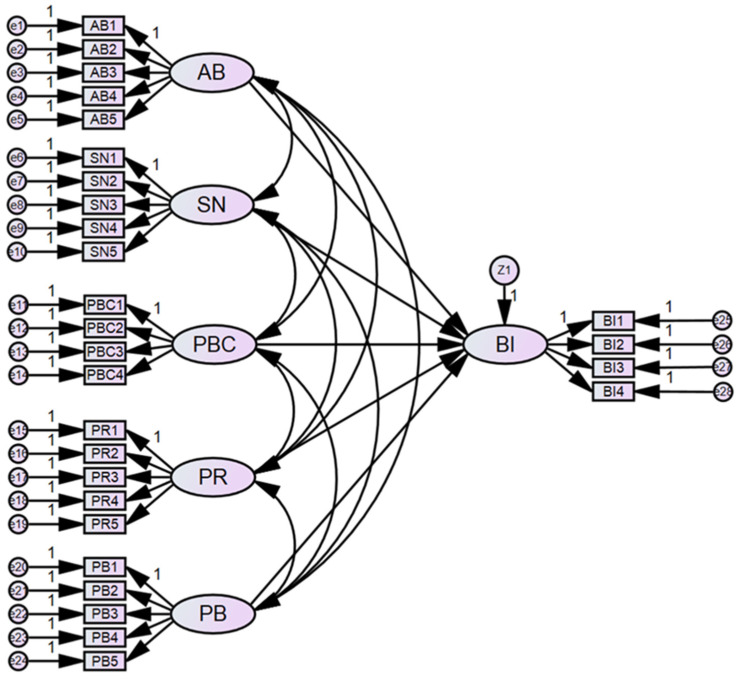
The initial structure model.

After measurement model was tested, the initial structure model was constructed, as shown in Figure 3. There are six constructs in the model, and each latent factor has several observed variables to be measured. The structural model was further tested by maximum likelihood method. The results of the initial model analysis are shown in Table 4 and Table 5. From Table 4, it can be seen that the initial model cannot fit the data well. Then, Table 5 indicates that some paths have insignificant *p*-values. Therefore, the initial model is necessary to be modified

**Figure 4:**
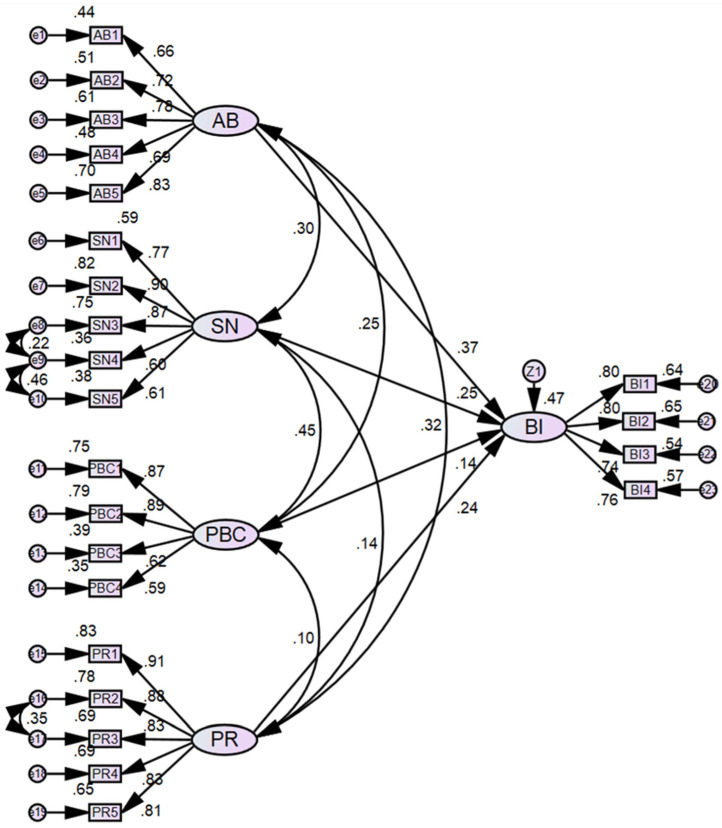
Standardized estimation of the final model.

To modify the initial model, it is essential to delete irrelevant paths. According to the results presented in Table 5, the path from PB to BI is not significant, having a p-value of 0.128. It generally means the hypothesis of H4 is rejected, i.e., perceived benefit cannot have a directly positive effect on public intention to participate in PPP projects. Thus, the constructor of PB was deleted to develop a new model. The model was revised by using the modification indices ultimately, and the final model is shown in Figure 4.

The results of the final model analysis are shown in Table 6 and Table 7. From Table 6, it can be concluded that the final model fits well with the data. From Table 7, it indicates that all paths are significant at 0.05 level respectively. As a result, the hypotheses of H1, H2, H3 and H5 are supported by the data. It revealed that attitude towards behavior (AB), subjective norm (SN), perceived risk (PR) and perceived behavioral control (PBC) are four main driving factors affecting public intention to participate in PPP projects.

## 6. Discussions

### 6.1. In-Depth Analysis of SEM Results

From the results, it is surprising that H4 is not supported, because it was regarded as a significant determinant for consumer behavior [68,108]. However, from this study, perceived benefit is regarded as an insignificant factor affecting public intention to participate in PPP projects. This may be because the public is not aware that they are the direct consumers of PPP projects. Besides this, the benefits brought by various PPP projects, such as employment, tourism and housing prices, will not be realized immediately, or it may be that public sectors exaggerate the publicity of the projects. When the public lack a real sense of experience, the potential benefits may be limited to them. In addition, it could be understood that the public understanding and participation in PPP projects are not enough, and their ability to perceive interests is also limited. When personal expectation is inconsistent with the goal of realizing public interests, the behavioral intention to participate in PPP projects would not be strong.

The hypothesis that perceived risk has direct effect on behavioral intention is supported. Indeed, when the public realize that PPP projects may harm their economic, health or environmental benefits during construction or operation, such emotions would provoke a positive attitude of the public to participate in projects decision making or supervision, and thus drive the public to generate willingness to participate. For example, in PPP projects of the NIMBY facilities, the public may be aware of the risks and health hazards of environmental pollution when the construction information of the project is disclosed or ready to be launched. At this point, the public are willing to participate in decision making to reduce risks. Instead, public opposition could hinder the project. Some studies have shown that the public had great opinions on the NIMBY facility projects [109,110]. Therefore, the government should not only properly publicize the original intention of PPP to realize the public benefits, so that the public can truly understand and participate in PPP projects, but also fully disclose the potential risks of the projects, and consult with the public to solve problems in order to gain more understanding and support from the public.

In terms of total effect on public intention to participate in PPP projects, attitude towards behavior played a greatest role amongst all predictors. This is aligned with findings of Turcanu et al. (2014), who revealed that attitude towards behavior was the most important influencing factor of public participation. This may be because attitude, as a comprehensive psychological reaction tendency, is the internal driving force of willingness. When the public understand the essence of PPP projects and the benefits of public participation, the sense of participation would be recognized at the level of public attitude, thus laying a foundation for their intention to participate in PPP projects. According to the results, for the sake of improving the attitude towards public participation in PPP projects, we can firstly strengthen the publicity of PPP and public participation, establish a reasonable incentive mechanism and drive public participation from the perspective of interests. Furthermore, relevant government departments should show their sincerity of paying attention to public opinions and accepting supervision, so as to cultivate public awareness of social responsibility consciousness.

As for the other two TPB predictors, subjective norm and perceived behavioral control also significantly influenced behavioral intention. Therefore, several managerial implications could be put forward from these results. For example, the government need to strengthen their credibility and play a leading role; the media need to meet more public interests demands and information requirements, and form a good public opinion environment. Non-governmental organizations such as community neighborhood committees should be a bridge between the government and the public, guiding the collective participation of community residents. Furthermore, in order to improve the perceived behavioral control, relevant government departments can enhance the education of public participation knowledge and ability; at the same time, the government should strengthen the degree of information disclosure, broaden the channels of public participation, and improve the corresponding legal system.

### 6.2. Public Preferences of Participation Approaches

In previous studies, scholars have pointed out that participation approaches could also significantly affect the public participation [111,112]. This view is demonstrated in the factor of perceived behavioral control in this study. When the public have more suitable approaches to choose, their intention to participate in PPP projects would be stronger. In this research, eight public participation approaches were identified from the literature to investigate the respondents’ preferences for participation approaches in PPP projects [38,113,114,115,116]. The analysis results are shown in Table 8. From Table 8, it can be seen that the most preferred approach of public participation is internet platform. This is because the internet platforms can provide a more convenient and time-saving approach to the public. Thus, in order to encourage more public participation in PPP projects, it is suggested to utilize more internet platforms, and try to introduce emerging technologies like virtual reality on these platforms. In addition, the information disclosure or consultation provided by the government is also a favorable approach to the public. By taking the initiative to publicize the information and solve the questions about PPP projects through various channels, government departments can cultivate public trust so as to facilitate sustainable development of PPP projects.

## 7. Conclusions

Public participation is of great significance to the sustainable development of PPP projects. However, the effectiveness and enthusiasm of public participation in PPP projects are not high at present. In order to improve the public intention to participate in PPP projects, this study used the TPB model to explore the driving factors of public intention to participate in PPP projects. The results showed that the additional factor “perceived benefit” had a limited influential impact on the public intention, whereas the effect of additional factor “perceived risk” was very significant. Of the three TPB predictors, attitude towards behavior had a greater impact than subjective norm and perceived behavioral control. Based on these findings, effective measures to improve public participation in PPP projects include: actively guiding the public to understand PPP projects; fully disclosing the potential risks of projects; and using virtual reality technology in Internet platforms. In terms of the existing literature, this study expanded TPB application in the public participation in PPP projects. In addition, two external factors were tested which expanded the classical TPB. The conclusions are useful to government officers relevant to PPP especially in developing countries so as to improve the public participation in PPP projects.

However, despite the contributions provided in this study, there are some limitations. First, the sample size used in this study is not ideally enough, which only meets the minimum requirement for the SEM. Second, the district of respondents could be more focused. Various cities in China may have different implementations of PPP projects; thus, the respondents may respond dissimilarly. Third, the data were mainly collected online, which may have impacts on the results related to the eight public participation approaches. Future research could be carried out in more specific districts which are with similar economic and social development status, and the questionnaire survey could be conducted with a larger sample size. In addition, if project resources (e.g., time, funding) are sufficient, on-site survey is suggested in order to eliminate possible bias caused by online survey.

## Figures and Tables

**Table 1:** Demographic characteristics of the respondents.

Characteristic	Distribution of Answers
Gender	Male: 48.4%; Female: 51.6%
Age	<20: 3.6%; 20–29: 76%; 30–39: 12.7%; 40–49: 5.4%; ≥50: 2.3%
Education level	PhD: 6.8%; Master: 31.7%; Bachelor: 52.5%; College or below: 5.4%
Workplace	Government department: 6.8%; Public institution: 12.2%; State-owned enterprise: 5.4%; Private enterprise: 21.7%; Others: 45.3%

**Table 2:** Testing coefficients in the measurement model.

Constructs	Items	Item Loadings	Cronbach’s α
AB	AB1	0.66 ***	0.857
	AB2	0.72 ***	
	AB3	0.79 ***	
	AB4	0.70 ***	
	AB5	0.83 ***	
SN	SN1	0.75 ***	0.879
	SN2	0.87 ***	
	SN3	0.89 ***	
	SN4	0.68 ***	
	SN5	0.66 ***	
PBC	PBC1	0.86 ***	0.827
	PBC2	0.88 ***	
	PBC3	0.63 ***	
	PBC4	0.63 ***	
	PBC5	0.43 ***	
PR	PR1	0.90 ***	0.932
	PR2	0.91 ***	
	PR3	0.87 ***	
	PR4	0.81 ***	
	PR5	0.79 ***	
PB	PB1	0.58 ***	0.834
	PB2	0.80 ***	
	PB3	0.84 ***	
	PB4	0.55 ***	
	PB5	0.77 ***	
BI	BI1	0.82 ***	0.855
	BI2	0.79 ***	
	BI3	0.74 ***	
	BI4	0.75 ***	

AB: Attitude towards Behavior; SN: Subjective Norm; PBC: Perceived Behavioral Control; PB: Perceived Benefit; PR: Perceived Risk; BI: Behavioral Intention; ***: *p* < 0.001.

**Table 3:** Goodness-of-fit of the measurement model.

Goodness-of-Fit Measure		Level of Acceptance Fit	Fit Statistics
Absolute fit	CMIN/DF	1~2 good	1.788
	GFI	>0.80 acceptable; >0.90 good	0.844
	AGFI	>0.80 acceptable; >0.90 good	0.808
	RMSEA	<0.10 acceptable; <0.08 good	0.060
Incremental fit	IFL	>0.90	0.930
	CFI	>0.90	0.929
Simple fit	PNFI	>0.50	0.746
	PGFI	>0.50	0.686

CMIN/DF: Chi-Square Fit Statistics/Degree of Freedom; GFI: Goodness-of-Fit Index; AGFI: Absolute Goodness-of-Fit Indices; RMSEA: Root Mean Square Error of Approximation; IFL: Incremental Fit Index; CFI: Comparative Fit Index; PNFI: Parsimony-Adjusted Measures Index; PGFI: Parsimony Goodness-of-Fit Index.

**Table 4:** Goodness-of-fit of the structure model.

Goodness-of-Fit Measure		Level of Acceptance Fit	Fit Statistics
Absolute fit	CMIN/DF	1~2 good	2.020
	GFI	>0.8 acceptable; >0.9 good	0.827
	AGFI	>0.8 acceptable; >0.9 good	0.790
	RMSEA	<0.1 acceptable; <0.08 good	0.068
Incremental fit	IFL	>0.9 good	0.908
	CFI	>0.9 good	0.907
Simple fit	PNFI	>0.5 good	0.738
	PGFI	>0.5 good	0.804

CMIN/DF: Chi-Square Fit Statistics/Degree of Freedom; GFI: Goodness-of-Fit Index; AGFI: Absolute Goodness-of-Fit Indices; RMSEA: Root Mean Square Error of Approximation; IFL: Incremental Fit Index; CFI: Comparative Fit Index; PNFI: Parsimony-Adjusted Measures Index; PGFI: Parsimony Goodness-of-Fit Index.

**Table 5:** Regression weights in the initial model.

			Estimate	S.E.	C.R.	*p*
BI	<---	AB	0.334	0.094	3.532	***
BI	<---	SN	0.262	0.078	3.361	***
BI	<---	PBC	0.119	0.062	1.931	0.053
BI	<---	PR	0.151	0.051	2.961	0.003
BI	<---	PB	0.176	0.116	1.523	0.128
AB1	<---	AB	1.000			
AB2	<---	AB	1.030	0.113	9.130	***
AB3	<---	AB	1.226	0.126	9.698	***
AB4	<---	AB	0.976	0.112	8.742	***
AB5	<---	AB	1.264	0.126	10.036	***
SN1	<---	SN	1.000			
SN2	<---	SN	1.208	0.090	13.496	***
SN3	<---	SN	1.165	0.088	13.245	***
SN4	<---	SN	1.007	0.103	9.803	***
SN5	<---	SN	0.898	0.095	9.504	***
PBC1	<---	PBC	1.000			
PBC2	<---	PBC	1.091	0.070	15.571	***
PBC3	<---	PBC	0.824	0.085	9.699	***
PBC4	<---	PBC	0.750	0.081	9.302	***
PR1	<---	PR	1.000			
PR2	<---	PR	1.125	0.054	20.922	***
PR3	<---	PR	1.040	0.056	18.545	***
PR4	<---	PR	1.038	0.063	16.389	***
PR5	<---	PR	0.978	0.062	15.761	***
PB1	<---	PB	1.000			
PB2	<---	PB	1.265	0.146	8.683	***
PB3	<---	PB	1.361	0.154	8.849	***
PB4	<---	PB	0.876	0.126	6.966	***
PB5	<---	PB	1.263	0.148	8.525	***
BI1	<---	BI	1.000			
BI2	<---	BI	0.985	0.080	12.325	***
BI3	<---	BI	1.048	0.093	11.223	***
BI4	<---	BI	0.988	0.086	11.449	***

AB: Attitude towards Behavior; SN: Subjective Norm; PBC: Perceived Behavioral Control; PB: Perceived Benefit; PR: Perceived Risk; BI: Behavioral Intention; ***: *p* < 0.001.

**Table 6:** Goodness-of-fit of the final model.

Goodness-of-Fit Measure		Level of Acceptance fit	Fit Statistics
Absolute fit	CMIN/DF	1~2 good	1.739
	GFI	>0.8 acceptable; >0.9 good	0.872
	AGFI	>0.8 acceptable; >0.9 good	0.837
	RMSEA	<0.1 acceptable; <0.08 good	0.058
Incremental fit	IFL	>0.9 good	0.948
	CFI	>0.9 good	0.939
Simple fit	PNFI	>0.5 good	0.760
	PGFI	>0.5 good	0.813

CMIN/DF: Chi-Square Fit Statistics/Degree of Freedom; GFI: Goodness-of-Fit Index; AGFI: Absolute Goodness-of-Fit Indices; RMSEA: Root Mean Square Error of Approximation; IFL: Incremental Fit Index; CFI: Comparative Fit Index; PNFI: Parsimony-Adjusted Measures Index; PGFI: Parsimony Goodness-of-Fit Index.

**Table 7:** Regression weights in the final model.

			Estimate	S.E.	C.R.	*p*
BI	<---	AB	0.393	0.082	4.772	***
BI	<---	SN	0.262	0.078	3.378	***
BI	<---	PBC	0.124	0.063	1.978	0.048
BI	<---	PR	0.182	0.049	3.701	***
AB1	<---	AB	1.000			
AB2	<---	AB	1.010	0.110	9.203	***
AB3	<---	AB	1.214	0.123	9.840	***
AB4	<---	AB	0.965	0.109	8.844	***
AB5	<---	AB	1.228	0.122	10.090	***
SN1	<---	SN	1.000			
SN2	<---	SN	1.230	0.087	14.134	***
SN3	<---	SN	1.114	0.084	13.315	***
SN4	<---	SN	0.872	0.100	8.764	***
SN5	<---	SN	0.825	0.091	9.029	***
PBC1	<---	PBC	1.000			
PBC2	<---	PBC	1.092	0.070	15.578	***
PBC3	<---	PBC	0.824	0.085	9.706	***
PBC4	<---	PBC	0.749	0.081	9.285	***
PR1	<---	PR	1.000			
PR2	<---	PR	1.084	0.055	19.660	***
PR3	<---	PR	0.989	0.058	16.958	***
PR4	<---	PR	1.050	0.063	16.630	***
PR5	<---	PR	0.986	0.062	15.926	***
BI1	<---	BI	1.000			
BI2	<---	BI	0.983	0.080	12.318	***
BI3	<---	BI	1.045	0.093	11.221	***
BI4	<---	BI	0.985	0.086	11.440	***
BI	<---	AB	0.393	0.082	4.772	***
BI	<---	SN	0.262	0.078	3.378	***
BI	<---	PBC	0.124	0.063	1.978	0.048
BI	<---	PR	0.182	0.049	3.701	***
AB1	<---	AB	1.000			
AB2	<---	AB	1.010	0.110	9.203	***

AB: Attitude towards Behavior; SN: Subjective Norm; PBC: Perceived Behavioral Control; PB: Perceived Benefit; PR: Perceived Risk; BI: Behavioral Intention; ***: *p* < 0.001.

**Table 8:** Eight approaches of public participation in PPP projects.

Approach	Average Value	Standard Deviation	Average Standard Error
Internet platforms, such as Weibo or WeChat	3.93	0.876	0.059
Information disclosure or consultation provided by the government	3.70	0.900	0.061
Discussion with experts or NGOs	3.44	0.978	0.066
Newspapers, magazines, television news or other traditional media	3.40	0.966	0.065
Public lectures on PPP Projects	3.27	0.927	0.062
Community residents’ committees	3.26	0.983	0.066
Writing letters, telephone calls or site visits	3.06	1.021	0.069
Assemblies or parades	2.73	1.132	0.076

## Data Availability

The data presented in this study are available on request from the corresponding author.

## Appendix A

**Table A1:** Measurement Items in the Formal Questionnaire.

Constructs	Code	Measurement Items
Attitude towards behavior (AB)	AB1	I think public participation can reduce the government’s decision-making mistakes in PPP projects
	AB2	I think public participation can improve the public understanding of PPP projects and help the projects go smoothly
	AB3	I think public participation can effectively monitor the behavior of the government and enterprises in PPP projects
	AB4	I think public participation helps the public opinions on PPP projects to be referenced or adopted by the government, thus guaranteeing the public rights
	AB5	I think public participation helps PPP projects to build and operate according to local conditions
Subjective norm (SN)	SN1	My family approves of my participation in PPP projects
	SN2	My neighbors or friends encourage me to participate in PPP projects
	SN3	Residents’ community committee encourages me to participate in PPP projects
	SN4	The government encourages me to participate in PPP projects
	SN5	News media supports me to participate in PPP project
Perceived behavioral control (PBC)	PBC1	I have enough time to participate in PPP projects
	PBC2	I have enough energy to participate in PPP projects
	PBC3	I have enough cognitive ability to participate in PPP projects
	PBC4	I have sufficient ways to participate in PPP projects
	PBC5	I can obtain relevant information to participate in PPP projects
Perceived benefit (PB)	PB1	PPP projects have a positive impact on local housing prices will affect whether I participate in PPP projects or not
	PB2	PPP projects have a positive impact on local employment will affect whether I participate in PPP projects or not
	PB3	PPP projects have a positive impact on local transportation will affect whether I participate in PPP projects or not
	PB4	PPP projects have a positive impact on local tourism will affect whether I participate in PPP projects or not
	PB5	PPP projects have a positive impact on local education will affect whether I participate in PPP projects or not
Perceived risk (PR)	PR1	PPP projects have the potential risk of environmental pollution will affect whether I participate in PPP projects or not
	PR2	PPP projects have the potential risk of physical health will affect whether I participate in PPP projects or not
	PR3	PPP projects have the potential risk of mental health will affect whether I participate in PPP projects or not
	PR4	PPP projects have the potential risk of damaging local culture will affect whether I participate in PPP projects or not
	PR5	PPP projects have the potential risk of charging the public unreasonably will affect whether I participate in PPP projects or not
Behavioral intention (BI)	BI1	I am willing to participate in the decision-making of PPP projects
	BI2	I am willing to participate in the supervision of PPP projects
	BI3	I hope to be involved in the early stages of PPP projects
	BI4	I will recommend people around me to pay attention to information about PPP projects

AB: Attitude towards Behavior; SN: Subjective Norm; PBC: Perceived Behavioral Control; PB: Perceived Benefit; PR: Perceived Risk; BI: Behavioral Intention.
